# A Look Ahead

**Published:** 1883-05

**Authors:** 


### A LOOK AHEAD.

The Independent Practitioner has not always sailed in quiet waters. The ambition of its founder was an honorable one, and he has for years labored to establish a journal that should be the exponent of a better practice, the representative of the highest possibilities of the dental profession. It has always been a clean journal, uncontaminated by isms or pathies. It was his highest desire that it should be the index of those who were treating oral diseases, the connecting link between the medical profession and her young specialty offspring. In the effort to accomplish this task he has sacrificed both money and health, and he now feels that the time has arrived when he must abandon the helm, and turn his attention to other pursuits. But he is loth to see a journal which has so far preserved its independence, and kept aloof from all commercial interests, become the organ of any dealer in dental materials, and still less can he bear the thought that this child of his anxiety should cease to have a place in the literature of his profession. Both mind and body, however, absolutely require a change. In this emergency he has appealed to certain well-known dentists, and they have come to his relief.

It is not, at the present time, either expedient or wise to announce the arrangements which have been made for the future of this journal. In the next issue we hope to be able to lay the whole matter before our readers. Suffice it to say, for the present, that for every dentist who loves his profession and desires its best good, we think a joyful surprise is in store, and that if present projects do not miscarry, a field of usefulness opens before The

INDEPENDENT PRACTITIONER, which will be the fruition and accomplishment of all the hopes of its founder. It will be placed upon a secure basis, and beyond the chances of financial disaster. It will be sure of an abundance of excellent matter for publication, and will be certain and regular in its appearance.

While no longer distinctively a medical journal, it will contain much that will be especially interesting to medical men, and it hopes for an increased patronage from the parent profession. Until the projected scheme is accomplished it will be managed and conducted by the present editor, to whom all communications should be addressed.

